# LncRNA DANCR promotes proliferation and metastasis in pancreatic cancer by regulating miRNA‐33b

**DOI:** 10.1002/2211-5463.12732

**Published:** 2019-12-10

**Authors:** Yongyun Luo, Qi Wang, Lili Teng, Jie Zhang, Jianjun Song, Wenping Bo, Di Liu, Yaqin He, Airong Tan

**Affiliations:** ^1^ Department of Hepatobiliary Surgery General Hospital of Ningxia Medical University Yinchuan China; ^2^ Department of Geriatric Medicine Shanghai East Hospital Tongji University School of Medicine Shanghai China; ^3^ Medical Experimental Center General Hospital of Ningxia Medical University Yinchuan China; ^4^ Third Department of Oncology Qingdao Municipal Hospital (East Campus) Qingdao China

**Keywords:** cell proliferation, lncRNA DANCR, metastasis, miR‐33b, pancreatic cancer

## Abstract

Increasing evidence indicates that long noncoding RNAs (lncRNAs) function as important regulators in biological processes and are dysregulated in various tumors. The lncRNA DANCR functions as an oncogene in various cancers, but elucidation of its role in pancreatic cancer (PC) requires further investigation. In the current study, we demonstrate that DANCR was increased in PC tissues and cell lines. Knockdown of DANCR significantly suppressed cell proliferation, migration, and invasion and influenced the levels of epithelial‐to‐mesenchymal transition‐associated proteins, as demonstrated by the observation of enhanced E‐cadherin levels and reduced N‐cadherin levels in PC cells. In addition, we identified direct binding to the predicted miR‐33b binding site on DANCR. We also showed that there is reciprocal repression between DANCR and miR‐33b. Furthermore, a miR‐33b inhibitor partially abrogated knockdown of DANCR and caused inhibitory effects. We also demonstrated that DANCR functions as a miR‐33b sponge to positively regulate MMP16 expression in PC cells. Collectively, the data reveal that DANCR exerts its function by regulating miR‐33b/MMP16 expression, implying an important role for a lncRNA–miRNA–mRNA functional network and suggesting a novel potential therapeutic target for PC.

## Graphical abstract

Long noncoding RNAs commonly exert their biological effects by functioning as sponges for miRNAs to counteract miRNA‐mediated mRNA suppression. Upregulation of lncRNA DANCR suppressed miR‐33b expression by binding with the miR‐33b, thereby increasing the expression of the miR‐33b target MMP16, and promoting PC migration and invasion. Our results reveal that DANCR promotes PC progression by functioning as a miR‐33b sponge to positively regulate MMP16 expression.

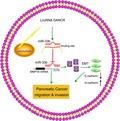

**Abbreviations**
DANCR‐Mt: mutant DANCR
DANCR‐Wt: wild‐type DANCR
ECL: electrochemiluminescent
EMT: epithelial‐to‐mesenchymal transition
lncRNAs: long noncoding RNAs
MTT: 3‐(4,5‐dimethyl‐2‐thiazolyl)‐2,5‐diphenyl‐2‐H‐tetrazolium bromide
PC: pancreatic cancer
SD: standard deviation
shDANCR: short‐hairpin RNA targeted DANCR

Pancreatic cancer (PC) is one of the deadliest malignancies in the world and is an aggressive cancer with almost uniform lethality 1. Approximately 40 000 patients die from PC each year, and patients with PC have poor prognosis and clinical outcomes, with a 5‐year survival rate of 6–7% 2. Surgical excision is the most effective treatment option for PC, but merely 9% of PC patients undergo this procedure 3. PC cell migration and invasion are the primary elements leading to poor prognosis 4. An investigation of the mechanisms underlying PC pathogenesis is needed to aid the identification of markers that are helpful in developing innovative diagnostic and therapeutic methods for PC treatment.

Long noncoding RNAs (lncRNAs) are noncoding RNAs (> 200 nt) with diverse and largely uncharacterized biological functions 5, 6. Emerging evidence indicates that lncRNAs participate in cellular processes, including proliferation, migration, and apoptotic processes, which are important in the development of cancer 7, 8. Previous studies have reported that lncRNAs function as oncogenes or tumor suppressors and are linked to cancer initiation and development, and lncRNAs have been identified as future diagnostic, therapeutic, or prognostic cancer biomarkers, including for PC. For example, lncRNA XLOC_000647 inhibited PC progression and reduced epithelial‐to‐mesenchymal transition (EMT)‐induced cell invasion by repressing NLRP3 9, and high expression of lncRNA CCDC26 could be a useful biomarker for tumorigenesis in PC 10. The lncRNA MALAT1 accelerated aggressive PC proliferation and metastasis through the stimulation of autophagy 11. These findings indicate that lncRNAs could be vital regulators during tumorigenesis and tumor progression.

The lncRNA DANCR (differentiation antagonizing nonprotein coding RNA) (NONCODE ID: NONHSAG037936.2) is located at human chromosome 4q12 and has been shown to be upregulated in various malignant tumors 12, 13. Mao *et al*. 14 showed that lncRNA DANCR induced invasion and migration by suppressing lncRNA‐LET in gastric cancer. Wang *et al*. and Jiang *et al*. 15, 16 reported that lncRNA DANCR works as an oncogene and promotes osteosarcoma proliferation and metastasis. Moreover, upregulated expression of lncRNA DANCR was found in hepatocellular carcinoma and DANCR action enhanced stemness features, offering a prognostic biomarker for hepatocellular carcinoma 17. These studies indicate that DANCR has an oncogene function in various cancers. Therefore, the role and biological mechanism of DANCR in PC require exploration.

In the current study, we showed that DANCR levels were enhanced in PC cell lines and tissues. Downregulation of DANCR remarkably suppressed PC cell viability and invasion by negatively regulating the expression of miR‐33b. In conclusion, our data suggest that the lncRNA DANCR promotes PC progression and is a potential therapeutic target for PC intervention.

## Materials and methods

### Human tissue specimens and cell culture

pancreatic cancer tissues (30 pairs of PC tumor samples and their corresponding nontumorous tissues) from patients who underwent surgical resection at General Hospital of Ningxia Medical University were collected. None of the patients had received radiotherapy or chemotherapy before surgery. Written informed consent was obtained from each patient, and the experimental protocol was authorized by the Ethics Committee of General Hospital of Ningxia Medical University. A total of five PC cell lines (AsPC‐1, PANC‐1, CFPAC‐1, SW1990, and BxPC‐3) and the normal human pancreatic epithelial cell line HPDE6‐C7 were obtained from the Shanghai Cell Bank, Type Culture Collection Committee of the Chinese Academy of Science (Shanghai, China). The cells were cultured in Dulbecco's modified Eagle's medium or RPMI 1640 medium (containing 10% FBS) (Gibco, Grand Island, NY, USA). The study methodologies conformed to the standards set by the Declaration of Helsinki.

### Quantitative real‐time polymerase chain reaction (qRT‐PCR) analysis

Total RNA was obtained from tissues and cells using TRIzol reagent in accordance with the manufacturer's instructions. cDNA was synthesized using the PrimeScript RT Reagent Kit (Takara, Dalian, China). qRT‐PCR was performed using SYBR Premix Ex Taq II (Takara). Glyceraldehyde‐3‐phosphate dehydrogenase and small nuclear RNA U6 were normalized as endogenous controls. The $2^{-\Delta \Delta C_{t}}$ method was performed to analyze relative expression.

### Oligonucleotide, plasmid construction, and cell transfection

miR‐33b mimics, miR‐33b inhibitor, and their negative controls were obtained from RiboBio (Guangzhou, China). To suppress the expression of DANCR, sequences of short‐hairpin RNA targeted DANCR (shDANCR) were constructed in a U6/GFP/Neo plasmid (GenePharma, Shanghai, China). The sequence used for shDANCR is as follows: 5′‐GGAGCTGAACTGCACTGTTGT‐3′. The plasmid carrying a nontargeting sequence was transfected and served as a control (shNC). Transfections were performed using the Lipofectamine 2000 reagent. At 48 h after transfection, stably transfected cells were selected using culture medium containing 0.5 mg·mL^−1^ of G418 (Sigma‐Aldrich, St Louis, MO, USA). After approximately 4 weeks, G418‐resistant cell clones were established.

### Dual‐luciferase reporter assay

A pmirGLO luciferase expression vector (Promega, Madison, WI, USA) was used to construct the reporter plasmid. A wild‐type DANCR (DANCR‐Wt) reporter plasmid was cloned by inserting the fragment from DANCR containing the predicted miR‐33b binding site. A mutant DANCR (DANCR‐Mt) reporter plasmid was created by mutating the seed region binding site of miR‐33b. HEK293T cells were plated in 6‐well plates and were cotransfected with a luciferase reporter vector containing DANCR‐Wt or DANCR‐Mt fragments and miR‐33b mimics or a negative control. After 48 h, luciferase activity was assayed.

### Cell viability assay

An MTT (3‐(4,5‐dimethyl‐2‐thiazolyl)‐2,5‐diphenyl‐2‐H‐tetrazolium bromide) assay was used to detect cell proliferation. Transfected PANC‐1 or SW1990 cells were seeded in 96‐well plates, and cell proliferation was measured at different time points (1, 2, 3, 4, and 5 d after seeding). Briefly, MTT solution (20 μL; Sigma‐Aldrich) was added to the cells for 4 h, the medium was removed, and then, dimethyl sulfoxide was added. The absorbance at 570 nm was determined using a Quant Universal Microplate Spectrophotometer (BioTek, Winooski, VT, USA).

### Colony formation assay

Cells were added to 6‐well plates (500 cells/well) after transfection. After 2 weeks, the cells were fixed, stained, and then imaged and counted using a light microscope.

### Cell migration and invasion assays

For migration assays, 2 × 10^4^ cells were added into the upper chamber of a transwell insert, and medium (10% FBS) was added to the lower chamber. For invasion assays, chamber inserts were precoated with BD Matrigel and medium overnight under sterile conditions. Then, 1 × 10^5^ cells were seeded in the upper chamber. After 24 h, cells on the top side of each insert were scraped off and fixed in methanol, and stained using crystal violet. Three random microscopic fields were counted for each group.

### Western blot assay

Total protein was obtained from the cells. Protein lysates were measured using the bicinchoninic acid assay method, and the lysates were electrophoresed through SDS/PAGE and transferred to polyvinylidene fluoride membranes, which were blocked, incubated with primary antibodies overnight, and then incubated with a corresponding horseradish peroxidase‐conjugated secondary antibody. Antibody binding was visualized using an electrochemiluminescent (ECL) substrate. The primary antibodies used were E‐cadherin, N‐cadherin, and β‐actin (Cell Signaling Technology, Beverly, MA, USA). Protein bands were determined using an ECL detection kit (ECL; Thermo Scientific, Rockford, IL, USA).

### Statistical analysis

Experimental data are presented as mean ± standard deviation (SD) and were assessed using Student's *t*‐test and one‐way ANOVA. Statistical analysis was performed using graphpad prism 5.0 (GraphPad Prism Software, GraphPad, San Diego, CA, USA). *P *<* *0.05 was considered to indicate statistical significance.

## Results

### DANCR expression is increased in PC tissues and cell lines

**Figure 1:**
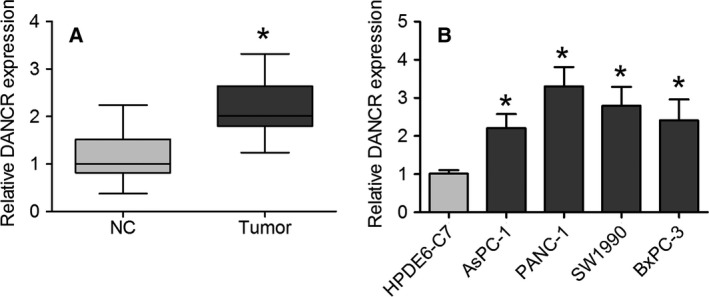
DANCR expression is increased in PC tissues and cell lines. (A) Expression of DANCR was measured using qRT‐PCR in PC tissues and healthy adjacent tissues. Data are expressed as mean ± SD, Student's *t*‐test. (B) qRT‐PCR analysis was used to determine DANCR expression in AsPC‐1, PANC‐1, CFPAC‐1, SW1990, BxPC‐3, and HPDE6‐C7 cell lines. Data are presented as mean ± SD of fold change, one‐way ANOVA. *P <* *0.05.

In the current study, the levels of DANCR were first determined in 30 pairs of PC tissues and healthy adjacent samples. As shown in Fig. 1A, the level of DANCR was higher in PC tissues than in noncancerous samples. DANCR expression was remarkably enhanced in the five PC cell lines compared with the HPDE6‐C7 cell line (Fig. 1B). These findings indicate that the upregulation of DANCR might participate in the progression of PC.

### Knockdown of DANCR suppresses PC cell proliferation

**Figure 2:**
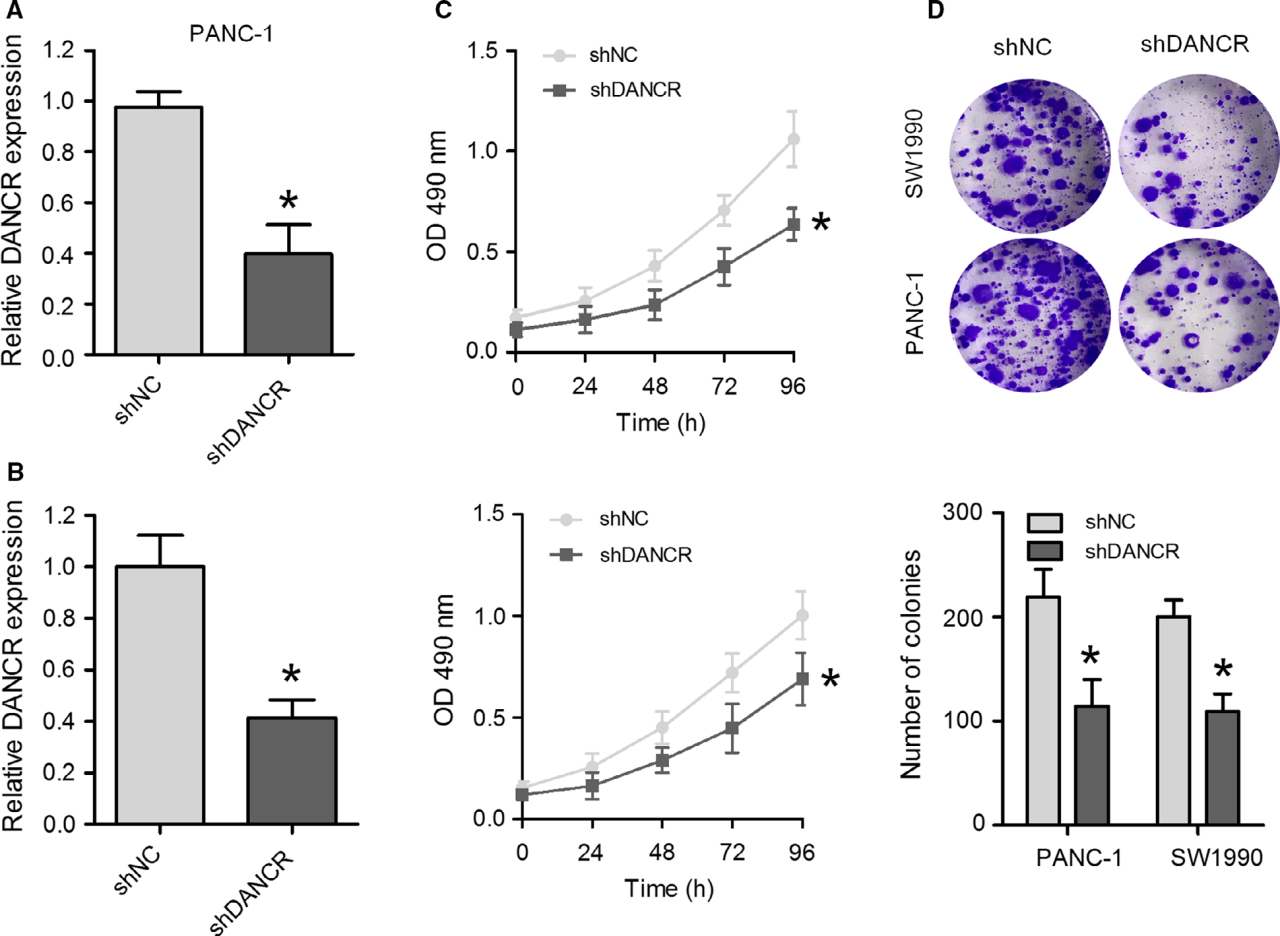
Knockdown of DANCR inhibits cell proliferation in PANC‐1 and SW1990 cells. DANCR expression was detected in PANC‐1 (A) and SW1990 cells (B) transduced with a DANCR shRNA vector (shDANCR) or negative control shRNA vector (shNC). (C) Cell viability was measured using an MTT assay at the indicated times after transfection. (D) Representative images (upper) and quantification (lower) of a colony formation assay in PANC‐1 and SW1990 cells transfected with shDANCR or shNC. Data are expressed as mean ± SD, Student's *t*‐test. *P <* *0.05.

To determine the potential biological role of DANCR in PC cells, two PC cell lines, PANC‐1 and SW1990, with higher expression of DANCR were chosen to assess the effects of shRNA‐mediated knockdown of DANCR on cell proliferation and colony formation. DANCR‐specific shRNAs (shDANCR) were evaluated for their knockdown efficiency, and we showed that shDANCR had a higher silencing efficiency compared with a negative control vector (shNC) (Fig. 2A,B). We observed that knockdown of DANCR caused a significant decrease in cell proliferation, as measured using MTT assays (Fig. 2C). DANCR knockdown also remarkably reduced colony formation (Fig. 2D). Collectively, these findings demonstrate that DANCR knockdown could suppress PC cell proliferation.

### Knockdown of DANCR inhibits PC cell metastasis

**Figure 3:**
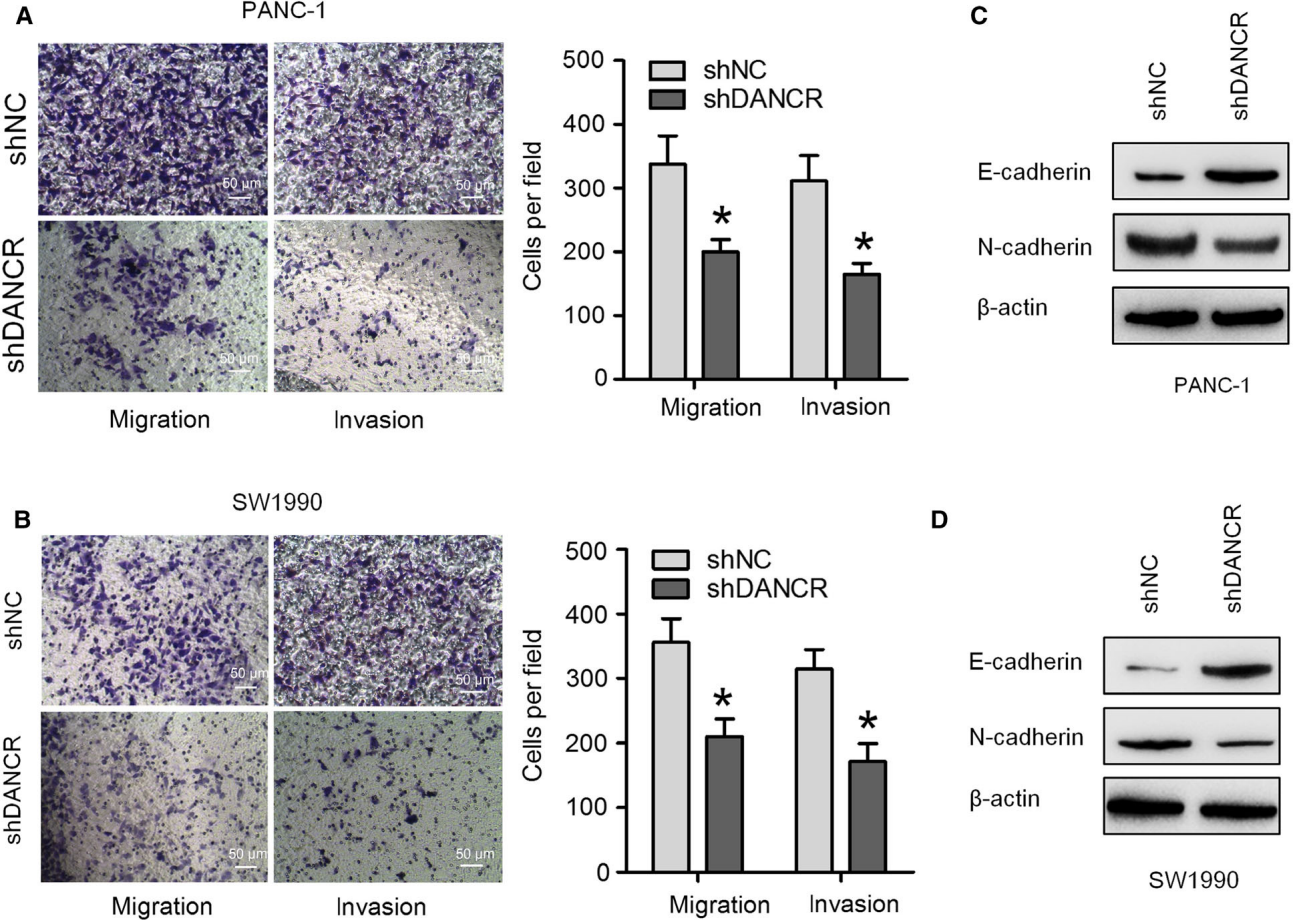
DANCR knockdown inhibits PC cell metastasis. Transwell migration and invasion assays were performed to assess cell migratory and invasive abilities in PANC‐1 (A) and SW1990 (B) cells transfected with shDANCR or negative control (scale bar = 50 μm). The protein expression of E‐cadherin and N‐cadherin in PANC‐1 (C) and BxPC‐3 (D) cells transfected with shDANCR or negative control was measured through a western blot assay. Data are expressed as mean ± SD, Student's *t*‐test. *P <* *0.05.

Next, we evaluated the role of DANCR in PC cell migration and invasion, which are critical factors in cancer progression and metastasis. Transwell migration assays revealed that knockdown of DANCR visibly suppressed the migratory activity of PANC‐1 and SW1990 cells when compared with the indicated controls (Fig. 3A,B). Transwell invasion assays demonstrated that the invasive capacities of PANC‐1 and SW1990 cell lines were dramatically inhibited by transfection with shDANCR, when compared with control cells (Fig. 3A,B). To determine whether the effect of DANCR on migration and invasion was mediated though EMT, a western blot assay was used to evaluate the levels of several EMT marker proteins in PC cells. The results demonstrated that DANCR knockdown caused a significant increase in E‐cadherin expression and resulted in a decrease in N‐cadherin expression (Fig. 3C,D). Taken together, these results suggest that downregulation of DANCR suppressed PC cell migration, invasion, and motility.

### DANCR negatively regulates miR‐33b expression in PC

**Figure 4:**
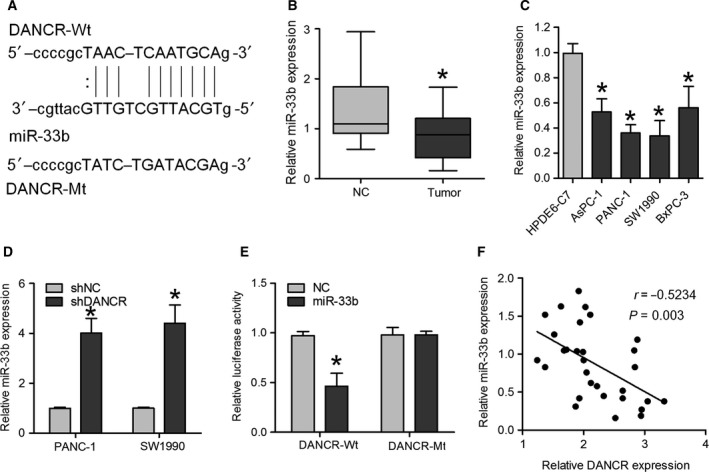
DANCR directly interacts with miR‐33b in PC cells. (A) Sequence alignment of miR‐33b with the putative binding sites within the wild‐type and mutant regions of DANCR. (B) Expression of miR‐33b was measured through qRT‐PCR in PC tissues and healthy adjacent tissues. Data are expressed as mean ± SD, Student's *t*‐test. (C) qRT‐PCR analysis was used to determine miR‐33b expression in AsPC‐1, PANC‐1, CFPAC‐1, SW1990, BxPC‐3, and HPDE6‐C7 cell lines. Data are expressed as mean ± SD, one‐way ANOVA. (D) miR‐33b expression was measured through qRT‐PCR in PANC‐1 and SW1990 cells transfected with shDANCR or shNC. Data are expressed as mean ± SD, Student's *t*‐test. (E) Relative luciferase activity of reporters containing DANCR‐Wt or DANCR‐Mt fragments in the indicated cells cotransfected with the indicated reporters and miR‐33b mimics or NC mimics. Data are expressed as mean ± SD, Student's *t*‐test. (F) The mRNA expression level of DANCR in 30 PC tissues was inversely related to miR‐33b expression. *P <* *0.05.

To explore potential molecular mechanisms, a target prediction program was used with the starBase v2.0 database to predict the possible targets of DANCR. Through the bioinformatics database, we found a seed sequence for miR‐33b in the DANCR mRNA (Fig. 4A), consistent with that reported in an earlier study 18. miR‐33b levels were lower in PC samples and cell lines than in noncancerous tissues and HPDE6‐C7 cells (Fig. 4B,C). To explore the effect of DANCR on miR‐33b expression, the level of miR‐33b was measured through qRT‐PCR in PANC‐1 and SW1990 cells following DANCR knockdown, and the results showed that miR‐33b levels were increased in PC cells transfected with shDANCR compared with control cells (Fig. 4D). In addition, we also demonstrated that DANCR expression was decreased in PANC‐1 and SW1990 cells transfected with miR‐33b (Fig. S1). Moreover, luciferase reporter vectors were constructed containing the DANCR‐Wt or DANCR‐Mt, and luciferase reporter assays were carried out to confirm whether miR‐33b is a direct target of DANCR. After cotransfection with miR‐33b mimics, the luciferase activity of the Wt 3′UTR reporter gene was dramatically reduced, but the activity of the Mt reporter gene was not changed by the miR‐33b mimics (Fig. 4E). In addition, a correlation analysis demonstrated a negative correlation between DANCR and miR‐33b in PC tissues (Fig. 4F). These findings suggest that DANCR negatively regulates miR‐33b expression in PC.

### The effects of DANCR in PC cells could be attenuated by miR‐33b

**Figure 5:**
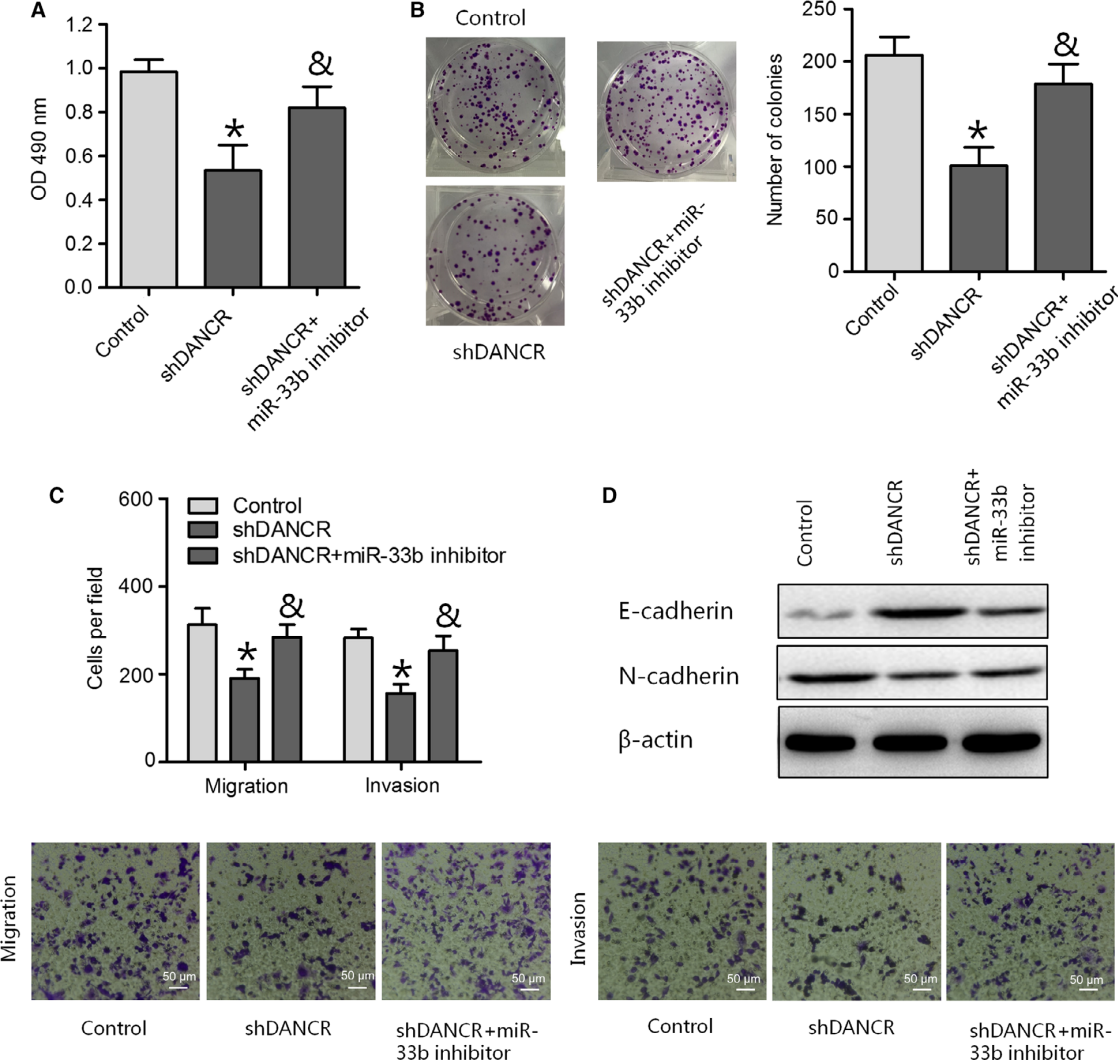
DANCR affects PC cell proliferation and metastasis by regulating miR‐33b expression. (A, B, and C) MTT, colony formation, and transwell migration and invasion assays were performed to determine PC cell viability and migratory and invasive abilities in PANC‐1 cells transduced with negative control or shDANCR or cotransduced with shDANCR and a miR‐33b inhibitor (scale bar = 50 μm). (D) The protein expression of E‐cadherin and N‐cadherin was analyzed through a western blot assay in PANC‐1 cells. Data are expressed as mean ± SD, one‐way ANOVA. *P <* *0.05 vs. control group; ^&^ *P *<* *0.05 vs. shDANCR group.

Given that DANCR can repress miR‐33b expression, we investigated whether miR‐33b could affect DANCR knockdown‐mediated inhibitory effects on PC progression. As expected, we observed that downregulation of miR‐33b expression abrogated the reduction in PANC‐1 cell proliferation and colony formation caused by shDANCR (Fig. 5A,B). The migratory and invasive abilities of PANC‐1 cells were remarkably enhanced after cotransfection with a miR‐33b inhibitor and shDANCR compared with cells transfected with shDANCR alone (Fig. 5C). Furthermore, a miR‐33b inhibitor could abrogate shDANCR‐induced E‐cadherin upregulation and N‐cadherin downregulation (Fig. 5D). These results suggest that downregulation of DANCR levels inhibited the proliferation and invasion of PC cells and EMT phenotypes by negatively modulating miR‐33b expression.

### DANCR positively regulates MMP16 expression via sponging miR‐33b

**Figure 6:**
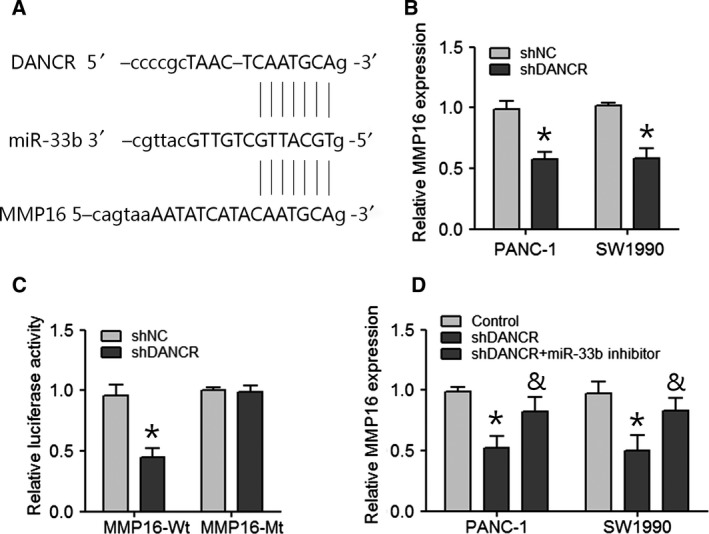
DANCR positively regulates MMP16 expression by sponging miR‐33b. (A) The putative miR‐33b‐binding sequence of DANCR and MMP16. (B) Expression of MMP16 was measured in shDANCR or shNC‐transfected PANC‐1 and SW1990 cells. Data are expressed as mean ± SD, Student's *t*‐test. *P <* *0.05. (C) Relative luciferase activity of HEK293T cells after cotransfection with either the MMP16‐Wt or MMP16‐Mt 3′UTR reporter gene along with either the shDANCR or shNC. Data are expressed as mean ± SD, Student's *t*‐test. *P <* *0.05. (D) Expression of MMP16 in PANC‐1 and SW1990 cells transfected with shDANCR or cotransfected shDANCR and miR‐33b inhibitor. Data are expressed as mean ± SD, one‐way ANOVA. *P <* *0.05 vs. control group; ^&^ *P *<* *0.05 vs. shDANCR group.

To further explore whether DANCR functioned as a miRNA sponge to positively regulate mRNA expression in a ceRNA‐dependent manner, we predicted miR‐33b target sites in an online bioinformatics database and predicted that DANCR and MMP16 mRNAs contain the same binding site for miR‐33b (Fig. 6A). We determined that MMP16 mRNA was significantly reduced in PANC‐1 and SW1990 cells transfected with shDANCR (Fig. 6B). In addition, a knockdown of DANCR decreased the luciferase activity of the cells transfected with MMP16‐Wt (Fig. 6C). PANC‐1 and SW1990 cells were transfected with the shNC, shDANCR, or shDANCR combined with miR‐33b inhibitor to investigate the effect of miR‐33b repression on MMP16 expression. MMP16 expression was decreased by shDANCR, and that effect was partially repressed by miR‐33b inhibitor (Fig. 6D). These results indicated that DANCR functioned as a miR‐33b sponge to positively regulate MMP16 expression in PC cells.

## Discussion

LncRNAs are involved in various cellular processes and are linked to the initiation and progression of malignant tumors 19. Here, we observed that DANCR levels were enhanced in PC tissues and cell lines. Downregulation of DANCR repressed PC cell viability and invasion. We also found that DANCR knockdown reverses EMT by enhancing E‐cadherin and decreasing N‐cadherin expression. Furthermore, we found that there was reciprocal repression between DANCR and miR‐33b expression, and this ensured the miR‐33b‐mediated tumor‐suppressive effects of DANCR in PC cells. We also demonstrated that DANCR functioned as a miR‐33b sponge to positively regulate MMP16 expression in PC cells. We demonstrated that downregulation of DANCR had tumor‐suppressive functions in PC cells by enhancing miR‐33b expression.

Increasing evidence suggests that lncRNA DANCR functions as a tumor promoter in numerous types of tumor. For example, remarkably high expression of DANCR was observed in patients with colorectal cancer, and overexpressed DANCR increased HSP27 levels and promoted proliferation and metastasis through miR‐577 sponging 13. DANCR was also upregulated in lung cancer 20, gastric cancer 21, triple‐negative breast cancer 22, and prostate cancer 23. Consistent with these reports, we observed that DANCR levels were higher in PC tissues and cell lines than in healthy tissues and HPDE6‐C7 cells, respectively, revealing that DANCR might act as an oncogene in PC.

To explore the effects of DANCR on the biological behaviors of PC cells, a series of biological experiments were performed. We showed that knockdown of DANCR significantly inhibited PC cell growth and proliferation. Furthermore, depletion of DANCR inhibited PC cells’ migratory and invasive abilities. These findings revealed that downregulation of DANCR had tumor‐suppressive effects in PC, suggesting that DANCR might contribute to PC progression.

Various studies have suggested that lncRNAs can function as ceRNAs, abolishing the endogenous suppressive effect of miRNAs on their targeted transcripts 24. Increasing numbers of researchers have proposed an interaction between lncRNAs and miRNAs in cancers 25, and we assumed that DANCR might function as a ceRNA in PC. To understand the mechanisms underlying the effects of DANCR in PC, bioinformatics analysis was used, and it revealed that a seed sequence of miR‐33b was present in the DANCR mRNA. miR‐33b has been observed to play an anti‐oncogene role in carcinogenesis. Previous studies have shown that miR‐33b prevented lung adenocarcinoma cell viability, invasion, and EMT by inhibiting the Wnt/β‐catenin/ZEB1 pathway 26. miR‐33b expression was also decreased in osteosarcoma 27, gastric cancer 28, lung adenocarcinoma 26, and breast cancer 29. Similarly, we determined that miR‐33b was also reduced in PC tissues and cell lines. In addition, we found that downregulation of DANCR significantly increased miR‐33b levels, and DANCR levels were decreased in PC cells transfected with miR‐33b mimics. Furthermore, we showed complementary pair binding between DANCR and miR‐33b. In addition, we further demonstrated that DANCR was negatively correlated with miR‐33b in PC tissues. These findings indicate that DANCR and miR‐33b may form a reciprocal repression feedback loop.

Furthermore, we explored whether the effects of DANCR in PC cells were affected by miR‐33b. Here, we revealed that a miR‐33b inhibitor rescued the suppressive effects that downregulation of DANCR had on PC cell viability and invasion. All our findings indicate that downregulation of DANCR inhibited PC cell viability and invasion through regulation of miR‐33b. However, the current study cannot exclude the possibility that DANCR might also affect other miRNAs or associated genes in PC, and this requires further investigation. In addition, we performed the online bioinformatics database and predicted that DANCR and MMP16 contain the same binding site for miR‐33b. Lin *et al*. 30 have reported that miR‐146b‐5p inhibits PC cell migration and invasion by targeting MMP16; thus, we speculated that DANCR may function as miR‐33b sponge to positively regulate MMP16 expression. In our study, we demonstrated that MMP16 expression was decreased by shDANCR, and that effect was partially repressed by miR‐33b inhibitor, which suggest that DANCR functioned as a miR‐33b sponge to positively regulate MMP16 expression in PC cells.

In summary, we observed that DANCR levels were remarkably reduced in PC samples and cell lines. Downregulation of DANCR expression reduced cell viability and invasion through upregulation of miR‐33b levels. These results suggest that DANCR is a key molecular marker in predicting prognosis and is an important target for PC therapy.

## Conflict of interest

The authors declare no conflict of interest.

## Author contributions

YYL and QW performed the experiments, and were responsible for data acquisition; they are co‐first authors of this study. LLT and JZ were responsible for data analysis and statistical analysis; they are co‐second authors of this study. JJS, WPB, and DL were responsible for data interpretation and visualization. YYL and QW were major contributors in writing the manuscript. YQH conceived and designed the study, and agreed to be accountable for all aspects of the work in ensuring that questions related to the accuracy or integrity of any part of the work are appropriately investigated and resolved. ART made significant contributions to interpretation of data and revised the manuscript critically for important intellectual content. All authors read and approved the final manuscript.

## Supporting information


**Fig. S1.** DANCR directly interacts with miR‐33b in PC cells. DANCR expression was detected using qRT‐PCR in PANC‐1 and SW1990 cells transfected with miR‐33b or its negative control. Data are expressed as mean ± SD, Student's *t*‐test. *P <* *0.05.
