# Supplementary figures and images for: LncRNA DANCR promotes proliferation and metastasis in pancreatic cancer by regulating miRNA‐33b
Source: FEBS Open Bio. 2019 Dec 10;10(1):18–27. doi: 10.1002/2211-5463.12732 (PMC6943224; doi:10.1002/2211-5463.12732)

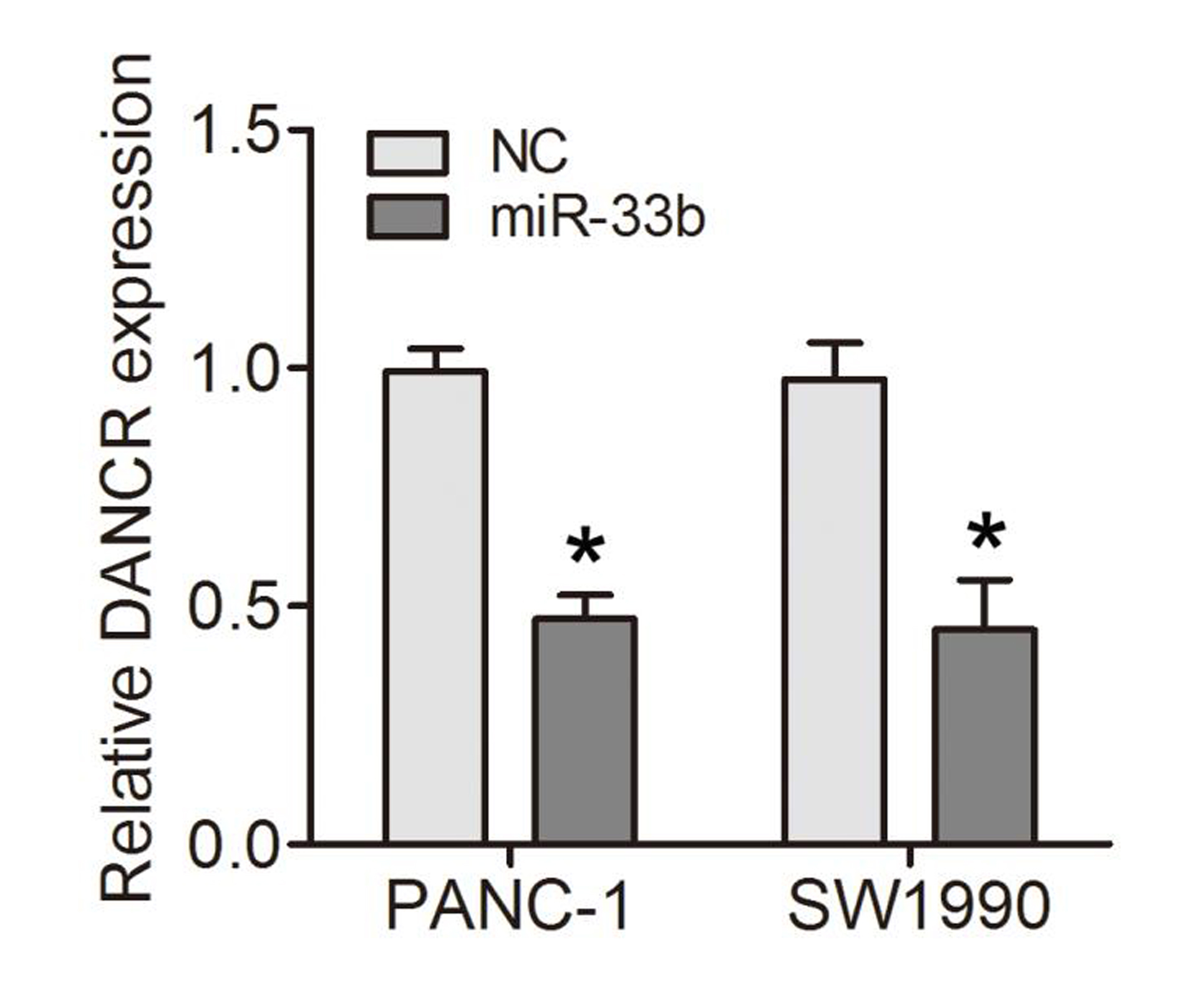

Supplement: Supplementary file 1 — Fig. S1. DANCR directly interacts with miR‐33b in PC cells. DANCR expression was detected using qRT‐PCR in PANC‐1 and SW1990 cells transfected with miR‐33b or its negative control. Data are expressed as mean ± SD, Student's t‐test. *P < 0.05. [file FEB4-10-18-s001.jpg]
